# Endoscopic transsphenoidal resection of sellar tumors with conchal sphenoid sinus: A report of two cases

**DOI:** 10.3892/ol.2014.2732

**Published:** 2014-11-24

**Authors:** YEXUN SONG, TIANSHENG WANG, JIANGBO CHEN, GUOLIN TAN

**Affiliations:** Department of Otolaryngology - Head and Neck Surgery, The Third Xiangya Hospital of Central South University, Changsha, Hunan 410013, P.R. China

**Keywords:** endoscopy, sellar region, transsphenoidal approach, sphenoid sinus

## Abstract

A conchal non-pneumatized sphenoid sinus was previously considered to be a contraindication to the endoscopic transsphenoidal route to the sella due to its small sellar floor and poor anatomical landmarks, such as the optic nerve canal, opticocarotid recess and internal carotid arteries canal. The present study aimed to investigate the methodology and characteristics of the endoscopic transsphenoidal resection of sellar tumors with a conchal sphenoid sinus. Two patients with sellar tumor patients and non-pneumatized sphenoid sinuses received endoscopic transsphenoidal surgery. The two conchal sphenoid sinuses were accessed safely, total resection was achieved and no serious complications occurred. Therefore, the presence of a conchal non-pneumatized sphenoid is not an absolute contraindication for employing the endoscopic transsphenoidal route in the resection of sellar tumors; a positive outcome may be achieved, in particular when the surgery is performed by an experienced otolaryngologist.

## Introduction

The endoscopic transsphenoidal route is considered the standard approach for the surgical resection of sellar tumors (1), which represent ~10% of all intracranial tumors, with a mortality rate of ~4.4% and a recurrence rate that varies between 6 and 21% (2). All of the various routes to the sella (transethmoidal, transnasal and transseptal), whether microscopic or endoscopic, pass through the sphenoid sinus to reach the sella. However, the presence of a conchal sphenoid sinus is typically considered to be one of the contraindications for the use of transsphenoidal route due to the difficulties of intraoperative localization and exposure of the sellar floor (3,4). The current study presents two cases of resection of sellar tumors with conchal sphenoid sinus via the endoscopic transsphenoidal route. Written informed consent was obtained from the patient’s family and the patient for case one and two, respectively.

## Case reports

### Case one

A 60-year-old female was admitted to the Department of Otolaryngology - Head and Neck Surgery of the Third Xiangya Hospital (Changsha, China) on August 28, 2011, presenting with a two-day history of a severe headaches, projectile vomiting and blepharoptosis of the left eye.

**Figure 1:**
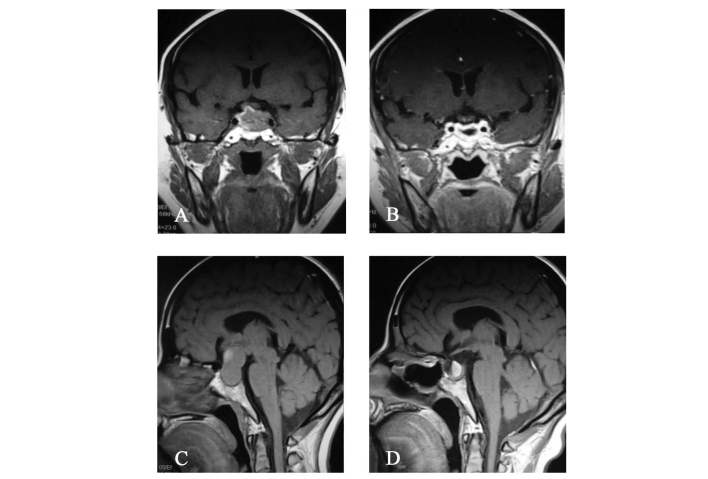
Case one: (A) Preoperative and (B) postoperative coronal MRI demonstrating the the total removal of a pituitary adenoma. Case two: (C) Preoperative and (D) postoperative sagittal MRI demonstrating the total removal of a pituitary adenoma. MRI, magnetic resonance imaging.

Nasal examination was normal. The right pupil size was 2.5 mm (normal range, 2.5–5.0 mm) with normal papillary reflex and the left pupil size was 4.0 mm with no papillary reflex. Visual acuity and visual field of the two eyes were normal. No strabismus or diplopia were observed and the eye movement was normal. Endocrinological investigations demonstrated decreased adrenocorticotropic hormone (ACTH; 1.10 pg/ml, normal range, 7.20–63.60 pg/ml) and luteinizing hormone (LH; 2.78 mIU/ml, normal range, 15.90–54.00 mIU/ml), normal thyroid stimulating hormone (TSH; 1.56 mIU/ml, normal range, 0.40–4.78 mIU/ml), follicle-stimulating hormone (FSH; 15.96 mIU/ml, normal range, 23.00–116.30 mIU/ml), growth hormone (GH; 0.43 ng/ml, normal range, 0.12–9.88 ng/ml) and prolactin (PRL; 2.48 ng/ml, normal range, 1.80–20.30 ng/ml) (Table I). Magnetic resonance imaging indicated a sellar tumor extended into the left cavernous sinus and a non-pneumatized sphenoid sinus (Fig. 1A). Following the aforementioned physical and clinical examinations, the tumor was excised under general anesthesia via the endoscopic transsphenoidal route. Skull base closure was achieved with a combination of autologous fascia lata grafts, fibrin glue and nasoseptal flap(s). The excised tumor was subjected to histopathology, which indicated a diagnosis of pituitary adenoma.

Postoperatively, the patient developed transient diabetes insipidus for six days, however no other complications, such as intracranial infection, cerebrospinal fluid leakage, intracerebral hemorrhage or cranial nerve palsies, were observed. Blepharoptosis recovered on day 13 after surgery. A magnetic resonance image captured one year following the surgery demonstrated that total resection was achieved (Fig. 1B). No recurrence was observed prior to the termination of follow-up, two years after the surgery was performed.

### Case two

A 45-year-old female presented with a three-month history of vision loss and was treated in the Department of Otolaryngology - Head and Neck Surgery of the Third Xiangya Hospital on December 3, 2013.

**Figure 2:**
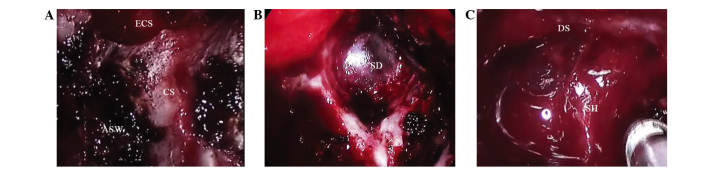
Intraoperative endoscopic view of endoscopic transsphenoidal excision of the sellar tumor in case two. (A) Anterior wall of sphenoidal sinus. (B) Exposure of the sellar dura. (C) Sellar region after total tumor resection. ECS, ethmoid cell superior to the sphenoid bone; CS, crista sphenoidalis; ASW, anterior sphenoidal wall; SD, sellar dura; DS, diaphragma sellae; SH, hypophysial.

The nasal examination was normal. The patient’s visual acuity (normal range, >1.0) was measured at 4 m using a retroilluminated logarithm of the minimum angle of resolution chart with tumbling-E optotypes (Precision Vision, La Salle, IL, USA), which revealed that the [visio oculus sinister (VOS)] in the left eye was counting fingers/30 cm and the [visio oculus dexter (VOD)] in the right eye was 0.3. Additionally, a visual field defect of bitemporal hemianopia was observed. Endocrinological investigations demonstrated elevated PRL (70.43 ng/ml, normal range, 1.80–20.30 ng/ml), decreased FSH (4.09 mIU/ml, normal range, 23.00–116.30 mIU/ml) and LH (0.39 mIU/ml, normal range, 15.90–54.00 mIU/ml), and normal TSH (3.29 mIU/ml, normal range, 0.40–4.78 mIU/ml), GH (0.31 ng/ml, normal range, 0.12–9.88 ng/ml) and ACTH levels (21.09 pg/ml, normal range, 7.20–63.60 pg/ml) (Table I). Magnetic resonance imaging indicated the presence of a sellar tumor, which had extended into the suprasellar cistern and caused compression of the optic nerves and optic chiasma, as well as a non-pneumatized sphenoid sinus (Fig. 1C). The surgical technique utilized in case two was identical to that used in case one; the intraoperative view is shown in Fig. 2. Subsequent histopathological examination of the lesion indicated a diagnosis of pituitary adenoma.

Postoperatively, the patient developed transient diabetes insipidus for 11 days. Prior to discharge (13 days following surgery), visual acuity was determined to be 0.8 VOS and 1.0 VOD, and visual field was fully recovered. Magnetic resonance imaging indicated that total resection of the tumor had been achieved, however, a hematoma was observed in the posterior pituitary fossa with no clinical symptoms (Fig. 1D). No additional complications were observed at the most recent follow-up examination in February 2014.

## Discussion

Sphenoid sinuses develop within the sphenoidal concha from the third embryonic month and may result in varying degrees of pneumatization in the corpora ossis sphenoidalis by the age of 14 years (5). According to the commonly used classification system proposed by Hammer and Radberg (6), the pneumatization of the sphenoid sinus is divided into three types: Conchal, presellar and sellar. The conchal type accounts for ~2% of patients who undergo surgery for sellar tumors (7) and the conchal non-pneumatized sphenoid was previously considered to be a contraindication to the transsphenoidal route to the sella due to its small sellar floor and poor anatomical landmarks, such as the optic nerve canal, opticocarotid recess and internal carotid arteries canal. Thus, the transsphenoidal approach is typically considered to be less favorable in cases of conchal non-pneumatized sphenoid sinus. In the present report, the two conchal sphenoid sinuses were accessed safely.

As determined in the present study, the key points with regard to the surgical procedure were as follows: i) Full use must be made of the preoperative imaging data, and the surgery-associated anatomical characteristics, as well as the depth and width of the sellar tumor requiring removal, must be determined; ii) the rostrum sphenoidale and posterior sections of the nasal septum must be used as the reference point for the midline, at which a position 2 cm above the choana atresia should be selected as the hypothetical position of the ostium of the sphenoidal sinus. Subsequently, the sphenoidal sinus should be entered via the hypothetical ostium; iii) a high-speed drill may be considered as an alternative to the osteotome and rongeur as it appeared to confer a number of advantages in the present case, including exerting a hemostatic effect on a small amount of bleeding from the sellar tumor, a clean surgical view and improved surgical safety; iv) lastly, in the event of bleeding, conventional procedures, such as curettage and suction, are often ineffective, and resection of the tumor may be achieved using a minimally invasive suction-dissection instrument, which was key in the success of the present surgical procedure (8).

In conclusion, for sellar tumors with non-pneumatized sphenoid, transcranial approaches are usually preferred by neurosurgeons, however, brain retraction and manipulation of neurovascular structures during the procedure may induce severe trauma and postoperative reactions, in addition to numerous complications which may increase the duration of the patient’s hospital stay. In the present study, a conchal non-pneumatized sphenoid does not appear to be an absolute contraindication for endoscopic transsphenoidal route in the resection of sellar tumors; endoscopic transsphenoidal surgery has a number of advantages when compared with other surgical approaches, including decreased morbidity, improved panoramic visualization and increased illumination and magnification. Furthermore, a positive outcome may be achieved, in particular when the surgery is performed by an experienced otolaryngologist. However, due to the small sample size used in the present study, future studies are required to confirm the efficacy and safety of the endoscopic route in the resection of sellar tumors with non-pneumatized sphenoid.

## Figures and Tables

**Table I:** Preoperative endocrinological investigations of the two cases.

Endocrinological investigation	Normal range	Case one	Case two
FSH	23.00–116.30 mIU/ml	15.96 mIU/ml	4.09 mIU/ml
GH	0.12–9.88 ng/ml	0.43 ng/ml	0.31 ng/ml
LH	15.90–54.00 mIU/ml	2.78 mIU/ml	0.39 mIU/ml
ACTH	7.20–63.60 pg/ml	1.10 pg/ml	21.09 pg/ml
PRL	1.80–20.30 ng/ml	2.48 ng/ml	70.43 ng/ml
TSH	0.40–4.78 mIU/ml	1.56 mIU/ml	3.29 mIU/ml

FSH, follicle stimulating hormone; GH, growth hormone; LH, luteinizing hormone; ACTH, adrenocorticotropic hormone; PRL, prolactin; TSH, thyroid stimulating hormone.
